# Pain threshold reflects psychological traits in patients with chronic pain: a cross-sectional study

**DOI:** 10.1186/s13030-017-0098-4

**Published:** 2017-05-12

**Authors:** Fumie Kato, Tetsuya Abe, Kenji Kanbara, Ikumi Ban, Tadashi Kiba, Sadanobu Kawashima, Yukie Saka, Yasuyuki Mizuno, Mikihiko Fukunaga

**Affiliations:** Department of Psychosomatic Medicine, Kansai Medical University, 2-5-1 Shinmachi, Hirakata-shi, Osaka Japan

**Keywords:** Chronic pain, Pain tolerance threshold, Central sensitization, High sensitivity, Minnesota Multiphasic Personality Inventory, Quantitative sensory testing

## Abstract

**Background:**

Chronic pain enhances sensory sensitivity and induces the biased development of psychological traits such as depression and pain catastrophizing, leading to the formation of heterogeneous conditions. Fluctuations in the sensory-related thresholds of non-injured sites (with normal peripheral tissue) in patients with chronic pain are thought to be related to central sensitization. The objectives of this study were to analyze the association between pain tolerance thresholds (PTTs) in non-injured sites and the psychological traits of patients with chronic pain and to evaluate the usefulness of PTT measures in assessments of pathological conditions related to chronic pain.

**Methods:**

This study included 57 patients with chronic pain. The PTTs were measured in non-injured sites with quantitative sensory testing (QST) with electrical stimulation and then classified with cluster analysis. The Short-Form McGill Pain Questionnaire was used to subjectively assess pain in the injured sites. The Minnesota Multiphasic Personality Inventory (MMPI) was used to assess the patients’ psychological traits.

**Results:**

Based on the cluster analysis of PTTs, the patients were classified into a High-Sensitivity group and an Others group consisting of the remaining patients. The results of the MMPI profiles showed that the High-Sensitivity group included significantly more patients with the Neurotic Triad pattern and no patients with the Conversion V pattern. The scores of the hypochondriasis and hysteria scales were significantly lower in the High-Sensitivity group than in the Others group.

**Conclusions:**

This study indicated that patients with chronic pain can be classified according to PTTs in non-injured sites and suggests that patients with High-Sensitivity have characteristic psychological traits. Assessment of PTTs in non-injured sites would be useful for evaluating the psychological condition of patients with chronic pain.

## Background

Pain is defined as an unpleasant sensory and emotional experience that is associated with actual or potential tissue damage or that is described in terms of such damage [1]. While assessments of both the associated sensations and emotions are important for understanding pain, these methods are limited because perception of pain differs among individuals and is affected by environmental and psychological factors at different times. In clinical practice, such traits related to pain make diagnosis and treatment difficult and contribute to the development of refractory and chronic pain [2, 3].

Chronic pain refers to pain of no fixed duration extending beyond the expected period of healing or to pain related to progressive non-cancer diseases [4]. As pathological conditions involve a combination of physical factors including tissue damage and psychosocial factors such as alexithymia [5], pain catastrophizing [6, 7], anxiety [8], living standard, and lifestyle [9], classification of these pathological conditions that are based on particular diseases or the affected tissues are not always useful for understanding the pathological conditions or determining appropriate treatments. No methods with common physiological or psychological indicators have been established to classify and evaluate patients with chronic pain.

The following three in vivo mechanisms transform acute pain into chronic pain: peripheral sensitization at the peripheral level, dysfunction of the descending pain inhibitory system, and central sensitization at the central level [10–13]. These mechanisms not only enhance pain at sites of tissue damage but also frequently induce the appearance of spontaneous pain or changes in the sensory sensitivity of non-injured sites to stimulation [8, 14, 15].

Quantitative sensory testing (QST) is a noninvasive method used to objectively assess subjective pain. Specifically, QST assesses neural function by quantifying the sensory-related thresholds of the responses of the examinees to various experimental stimuli, such as thermal, pressure, electrical, and ischemic stimuli [16–20]. The sensory-related thresholds at sites of injury are associated with the three in vivo mechanisms described above, whereas fluctuations in the sensory-related thresholds of non-injured sites reflect only mechanisms at the central level [17, 20]. Studies of the QST of chronic pain have shown that pain thresholds decrease at non-injured sites in female patients with non-traumatic neck-shoulder pain and patients with unilateral epicondylalgia [10, 21]. The results of these studies suggest that central sensitization causes fluctuations in the pain thresholds of non-injured sites in patients with chronic pain, regardless of their condition. Other previous studies suggest that QST is useful for classifying patients with chronic pain and for predicting treatment response [22–25].

Many studies have referred to the psychological traits of patients with chronic pain. These traits include alexithymia, catastrophizing, anxiety, and depression [5–7, 26–28]. Although it is easy to administer the self-reported questionnaires used to assess psychological traits in these studies, such as the Toronto Alexithymia Scale, the 36-item Short-Form Health Survey’s Mental Health Scale, and the Pain Catastrophizing Scale, the scales are based on subjective assessments performed by patients and do not include items regarding patient personality. The Minnesota Multiphasic Personality Inventory (MMPI) enables assessment of personality from various perspectives and can be used for screening for mental disorders. As this questionnaire includes validity scales, it is more likely to evaluate biased responses than do other types of questionnaires [29, 30]. The MMPI has been used to classify the psychological traits of patients with chronic pain, and characteristic MMPI profiles have been reported for these patients [31–34].

Factors that have been reported to be associated with low pain thresholds include physical variables, such as the severity and duration of the pain and decreased autonomic function [35, 36]. On the other hand, pain thresholds have been reported to increase in patients with depression [26–28]. Cruz-Almeida et al. classifyed patients with chronic pain by using psychological variables and displayed unique sets of clinical pain and somatosensory characteristics [37]. According to these reports, physical and psychological factors and which result in heterogeneous chronic pain is strongly associated with increasing or decreasing the pain thresholds and complicating the central sensitization.

Some patients with chronic pain are, however, resistant to psychological intervention [38, 39]. In order to evaluate the usefulness of pain threshold measures as a tool for assessing heterogeneous pathological conditions that involve chronic pain, this study aimed to clarify the association between pain thresholds in non-injured sites and the psychological traits of patients with chronic pain.

## Methods

### Participants

The participants were selected from 81 patients with chronic pain who had been admitted to the department of Psychosomatic Medicine of Kansai Medical University. Based on previous studies [40–42], the participants were diagnosed with nonmalignant chronic pain that had persisted for three or more months by attending physicians with clinical experience in treating chronic pain. The staff members of the Department of Psychosomatic Medicine are physicians, not psychiatrists, and the chief complaints of almost all patients are their physical symptoms. Patients with any of the following criteria were excluded: (1) an age of 18 years or less, (2) extensive peripheral neuropathy, (3) pain in the non-dominant hand, (4) opioid use, or (5) a diagnosis of major depression, schizophrenia, or dementia. After exclusions, the data of 57 patients with chronic pain was available for analysis. In accordance with the study protocol approved by the ethics committee of Kansai Medical University Hospital, written informed consent was obtained from the participants.

### Pain tolerance threshold (PTT) assessment

In this study, the QST was performed with a Neurometer CPT (Neurotron, Incorporated, Towson, MD, USA). With this device, an electrical stimulus of either 250 or 5 Hz was selectively applied to Aδ primary afferent fibers, which transmit sharp pain, and C primary afferent fibers, which transmit dull pain. The QST was performed in a quiet room at a comfortable temperature. The participants sat on a chair and a stimulating electrode was attached around the distal interphalangeal joint of the fourth finger of the non-dominant hand. The electrical stimulation current was increased from 0 to 9.99 mA at a set rate while the button on the device was pressed, and the current stopped when the button was released. After the patients were informed that stimulation with the highest electrical current would not cause tissue or other damage to the body, the participants then operated the device by themselves. They were instructed to release the button when the stimulus reached an unbearable level of pain, and this was defined as the PTT. The QST was performed once for each level of stimulation, and measurements were taken every 30 s. Previous studies have shown only a small level of variation among measurements [43–45].

### Assessment of pain intensity

Subjective assessments of the pain were performed with the Short-Form McGill Pain Questionnaire (SF-MPQ). The participants evaluated the pain with 15 expressions that described the sensations and the emotions of pain, which were rated on a 4-point scale, while the severity of the pain was evaluated with both a visual analog scale (VAS) and the Present Pain Intensity 6-point scale. The reliability of the Japanese version of the SF-MPQ has been confirmed [46].

### Psychological instrument

The MMPI questionnaires were distributed to the participants before the experiment and then collected at the time of the PTT measurement. The MMPI, which is a self-reported questionnaire on personality, is highly reliable for less invasively evaluating psychological traits from various perspectives. The MMPI consists of four validity scales (cannot say, lie, infrequency, and defensiveness) and ten clinical scales: Hypochondriasis (Hs), Depression (D), Hysteria (Hy), Psychopathic deviate, Masculinity/femininity, Paranoia, Psychasthenia, Schizophrenia, Hypomania, and Social introversion that are assessed with 550 questions that are answered on a 3 point scale (agree, disagree, and neither). The scores are calculated by assigning two points to agree and one point to neither. Higher scores indicate a greater tendency for that trait. The results are expressed as numerical values and profile forms [29, 30].

In Japan, the MMPI has been widely used in the clinical and academic fields [47–49]. In the United States, the transition to the MMPI-2 has already been completed. The basic scales of the MMPI are compatible with those of the MMPI-2, and the items considered important in the clinical and academic fields are common between the two versions. Thus, the assessment results are considered similar for the MMPI and MMPI-2.

### Statistical analysis

**Fig. 1:**
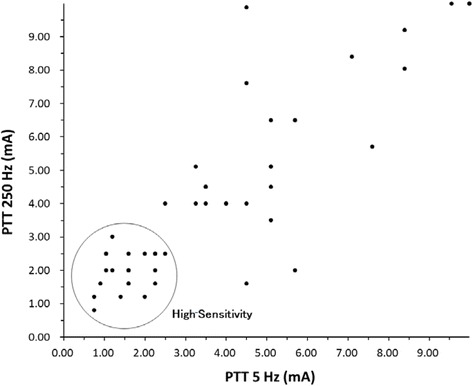
Scatter plot of the PTTs of the participants. Each circle represents one patient. A cluster analysis conducted with Ward’s method indicated that the Others group had higher PTTs than the High-Sensitivity group. PTT, pain tolerance threshold

All values are presented as mean ± standard deviation (SD). The statistical analyses were performed on the data of the 57 participants (22 male and 35 female). According to the scatter diagrams of the PTTs to the 250-Hz and 5-Hz stimuli (Fig. 1), the PTTs were not distributed evenly among the patients with chronic pain. Therefore, a cluster analysis (Ward’s method) was performed with PTTs as a variable of interest to extract a characteristic group. Inter group comparisons were performed with a *t*-test, one-way analysis of variance (ANOVA), χ^2^ test, or residual analysis. The SPSS software program (version 11.5, IBM Corporation, Armonk, NY, USA) was used for the analyses.

## Results

### Demographic and clinical characteristics of the patients

Of the participants, 61.4% were female. The mean age was 47.7 years, with a SD of 17.5 years. The mean pain duration was 59.3 months, with a SD of 75.0 months (Table 1). The main diagnosis was Chronic pain (40%), followed by Functional dyspepsia (10%), Fibromyalgia syndrome (9%), and Premenstrual syndrome (9%). The main sites of pain were the upper and low back (26%), the lower extremities (23%), the abdomen (16%), and the neck (14%) (Table 2). The SF-MPQ scores were 15.30 ± 7.78 for the sensory components and 6.00 ± 3.60 for the affective components, while current pain intensity was rated as 3.38 ± 1.21 on a six point scale ranging from 0 (no pain) to 5 (unbearable pain). The severity of pain at the injured site in the past week was rated as 6.71 ± 2.50 cm on a 10-cm VAS (Table 1).

**Table 1:** Patient characteristics

Variables	*n* = 57 (Female, 61.4%)	
	Mean	SD
Age (y)	47.75	17.51
Pain Duration (m)	59.33	73.00
250 Hz PTT (mA)	4.59	3.00
5 Hz PTT (mA)	4.23	2.93
SF-MPQ sensory score	15.30	7.78
SF-MPQ affective score	6.00	3.60
SF-MPQ Visual Analog Scale	6.71	2.50
SF-MPQ Present Pain Intensity	3.38	1.21

*SD* standard deviation, *PTT* pain tolerance threshold, *SF-MPQ* Short-Form McGill Pain Questionnaire

**Table 2:** The prevalence of each diagnosis and pain location

Diagnosis categories	%	Pain location	%
Chronic pain	40	Low back	26
Functional dyspepsia	10	Lower extremities (both/each)	23
Fibromyalgia syndrome	9	Abdomen	16
Premenstrual syndrome	9	Neck	14
Irritable bowel syndrome	4	Shoulder (both/each)	10
Spinal canal stenosis	4	Back	9
Herniated intervertebral disc, Splenic flexure syndrome, Combined headache, Tension headache, Chronic pancreatitis, Oral malaisis, Dysautonomia, Functional somatic syndrome		Epigastric region, Whole body, Half body, Head, Hypochondrial region, Buttocks, Chest, Upper arm(both/each)	

### PTT

The PTTs to 250-Hz stimulus ranged from 0.80 mA to 9.99 mA, with a mean ± SD of 4.59 ± 3.00 mA. The PTTs to 5-Hz stimulus ranged from 0.75 mA to 9.99 mA, with a mean ± SD of 4.23 ± 2.93 mA.

The mean ± SD PTTs to 250-Hz stimulus were 5.74 ± 3.19 mA for male and 3.87 ± 2.67 mA for female participants, while those to 5-Hz stimulus were 5.53 ± 3.19 mA and 3.41 ± 2.47 mA, respectively. The PTTs to both stimuli were significantly lower for female participants (250 Hz, *p* < 0.05; 5 Hz, *p* < 0.01).

### Cluster analysis of the PTTs

The cluster analysis done to create the High-Sensitivity group selected 23 participants with decreased PTTs (*n* = 23) (Fig. 1). The PTTs ranged from a minimum of 0.75 mA to a maximum of 3.00 mA. In contrast, the PTTs of the remaining participants were distributed in a wide range, from a minimum of 1.60 mA to a maximum of 9.99 mA. Since we thought it was not appropriate to describe the rest as a group with identical characteristics, this group was named Others (*n* = 34).

### Comparison of the demographic and clinical characteristics of the groups

Of the participants in the High-Sensitivity group 78.2% were female, as were 50.0% of the participants in the Others group. A χ^2^ test showed that the male-to-female ratio did not differ between the groups. The mean ± SD ages were 56.30 ± 15.83 years in the High-Sensitivity group and 41.94 ± 16.35 years in the Others group. The mean ± SD pain durations were 40.60 ± 76.42 months in the High-Sensitivity group and 73.23 ± 81.90 months in the Others group. The *t*-tests showed that neither age nor pain duration differed between the groups. In addition, *t*-tests showed that the scores on the sensitive and affective components of the SF-MPQ, VAS scores, and current pain ratings did not differ between the groups (Table 3).

**Table 3:** Characteristics of the high-sensitivity and the others groups

Variables	High-Sensitivity (*n* = 23)		Others (*n* = 34)		
	Female, 78.2%		Female, 50.0%		
	Mean	SD	Mean	SD	*p*
Age (y)	56.35	15.83	41.94	16.35	0.00**
Pain Duration (m)	40.61	76.48	73.23	81.90	0.10
250 Hz PTT (mA)	0.99	0.56	6.36	2.66	0.00**
5 Hz PTT (mA)	1.59	0.54	6.01	2.51	0.00**
SF-MPQ sensory score	13.91	7.98	16.29	7.61	0.27
SF-MPQ affective score	6.42	3.76	5.71	3.53	0.51
SF-MPQ Visual Analog Scale	7.12	2.36	6.43	2.60	0.32
SF-MPQ Present Pain Intensity	3.55	1.26	3.26	1.18	0.40

Test of significance by independent t-test

*SD* standard deviation, *PTT* pain tolerance threshold, *SF-MPQ* Short-Form McGill Pain Questionnaire

**p < 0.01

### Comparison of the MMPI clinical scales of the high-sensitivity and the others groups

**Fig. 2:**
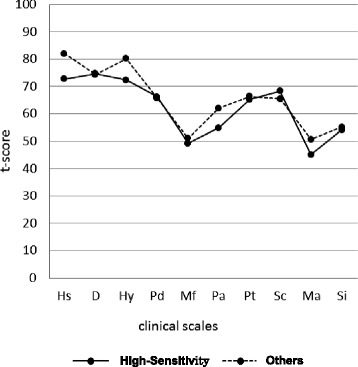
Line graphs of the *t*-scores for the clinical scales on the MMPI. The MMPI score profiles were compared between the High-Sensitivity (*n* = 23) and Others (*n* = 34) groups (one-way analysis of variance; Hs scale: F, 6.505; p, 0.014; Hy scale: F, 5.997; p, 0.018). MMPI, Minnesota Multiphasic Personality Inventory; Hs, Hypochondriasis; D, Depression; Hy, Hysteria; Pd, Psychopathic deviate; Mf, Masculinity-femininity; Pa, Paranoia; Pt, Psychasthenia; Sc, Schizophrenia; Ma, Hypomania; Si, Social introversion

In the MMPI profiles, both groups showed high values on the Hs, D, and Hy, scales (t ≥ 70), which are typical profiles for patients with chronic pain (Fig. 2). Patients were classified by three patterns of these three scales: Conversion V pattern, in which the scores on the Hs and Hy scales were higher than the score on the D scale by 10 or more points; the Neurotic Triad pattern, in which the scores on the Hs and Hy scales were lower than the score on the D scale; and the others patterns. The High-Sensitivity group contained no participants with the Conversion V pattern, 11 with the Neurotic Triad pattern, and 12 with other patterns, whereas the Others group contained 17, 7, and 10 participants in each of these categories, respectively (Table 4). A χ^2^ test (Yates’ correction) showed that the distribution of these patterns differed significantly between the groups. The residual analyses showed that the proportion of participants with the Conversion V pattern was significantly lower in the High-Sensitivity group, while the Neurotic Triad pattern was significantly higher (*p* < 0.01 for both). No significant difference in the proportions of the participants with the other patterns was observed.

**Table 4:** The number of subjects with the three Hs-D-Hy scale patterns

Hs-D-Hy scale patterns	Group	
	High-sensitivity (*n* = 23)	Others (*n* = 34)
Conversion V pattern (%)	0 (0)	17**(100)
Neurotic Triad pattern (%)	11** (61)	7 (39)
Other patterns (%)	12 (56)	10 (45)

Test of significance by χ^2^ test (Yates’ correction)

*Hs* hypochondriasis, *D* depression, *Hy* hysteria

**p < 0.01

Moreover, the scores on the MMPI clinical scales were compared with a one-way ANOVA. The scores on the Hs and Hy scales were significantly lower in the High-Sensitivity group than in the Others group (*p* < 0.05 for both) (Table 5).

**Table 5:** Comparison of the MMPI clinical scales

MMPI clinical scales	Group					
	High-sensitivity (*n* = 23)		Others (*n* = 34)			
	Mean	SD	Mean	SD	F	*p*
Hs	72.74	11.42	81.91	14.49	6.500	0.014*
D	74.70	13.58	74.38	10.80	0.009	0.923
Hy	72.43	12.30	80.18	11.29	5.997	0.018*
Pd	66.17	11.30	65.71	11.60	0.023	0.881
Mf	49.22	8.06	51.12	11.64	0.446	0.507
Pa	54.83	9.55	61.97	15.68	3.806	0.056
Pt	65.26	14.36	66.35	14.18	0.080	0.778
Sc	68.30	11.18	65.50	15.84	1.845	0.180
Ma	45.00	8.58	50.68	12.00	3.821	0.056
Si	54.09	10.56	55.15	11.32	0.127	0.723

Test of significance by one-way analysis of variance

*Hs* hypochondriasis, *D* depression, *Hy* hysteria, *Pd* psychopathic deviate, *Mf* masculinity-feminity, *Pa* Paranoia, *Pt* psychasthenia, *Sc* schizophrenia, *Ma* hypomania, *Si* social isolation

*p < 0.05

## Discussion

This study aimed to examine the relationship between the PTTs and psychological traits of patients with chronic pain in order to evaluate the usefulness of pain thresholds for assessing the pathological conditions of patients with chronic pain.

In this study, the PTT, which is an indicator of central sensitization, was measured in non-injured sites in patients with chronic pain. This study had two important findings: these measurements differentiated a High-Sensitivity group of patients who showed substantial decreases in their PTTs, and assessment with the MMPI revealed psychological traits that were characteristic of the patients with chronic pain, some of which have been reported by previous studies.

In this study, the PTT was significantly lower in female participants. Several previous studies on sex difference have shown that pain sensitivity differs between males and females. For instance, Fernández-Carnero et al. studied hyperalgesia in patients with unilateral epicondylalgia and reported that the pain threshold for experimental pain was lower in female than in male patients [21], and Kindler et al. studied the sensitivity to experimental and clinical pain in patients with shoulder pain and reported that females were more sensitive to both types of pain [50]. Our results supported these previous results.

Age was significantly higher in the High-Sensitivity group compared to that in the Others group. Previous studies have shown that aging is associated with an increase in pain perception threshold and a decrease in PTT, and this has been attributed to aging-induced impairments in both the excitatory and inhibitory functions in the mechanisms underlying pain perception [51]. The results obtained in the present study were similar to the results of these studies.

Although the pain duration of the participants did not differ between the High-Sensitivity and the Others groups, the pain duration varied widely, from 3 months to 240 months. While pain duration has been reported to be longer in patients with the Conversion V pattern [52], our results showed that some patients with long pain duration were in the Others group, which included many patients with the Conversion V pattern. An inverse correlation between pain duration and pain thresholds has also been reported [20]. Further studies are necessary to determine the association between pain duration and various factors.

### Classification of patients with chronic pain according to PTT

In this study, patients with chronic pain were classified using cluster analysis of the PTT at non-injured sites and their psychological traits were evaluated. As a result, we identified a characteristic High-Sensitivity group. Several previous studies done to classify patients with chronic pain have suggested a relationship between pain and psychological traits. Murphy et al. showed that there were multiple detectable subgroups of patients with chronic pain by cluster analysis of clinical pain intensity and psychological variables [53]. In addition, Cruz-Almedia et al. classified patients with chronic pain by cluster analysis of psychological variables and clarified the association between psychological characteristics and both clinical pain intensities and pain thresholds [37].

Previous studies were classified according to psychological variables. In this study, we classified patients with chronic pain by cluster analysis with only reproducible PTT as a physical variable to assess the relationship between psychological factors and PTTs. The results strongly supported the study of Cruz-Almedia et al., which showed close relationship among psychological traits, somatosensory sensations, and central sensitization to chronic pain [37]. This shows that patients with chronic pain can be classified by PTT as a physical variable.

### Association between PTT and psychological traits

This study yielded results indicating an association between classifications based on PTTs and psychological traits. Previous studies that used MMPI for patients with chronic pain found that their participants could be classified into three to six types [54–56], and the inclusion of the following three patterns is common to all of these classifications: the Conversion V pattern, the Neurotic Triad, and the normal patterns, which show scores within the normal range on all scales. The Conversion V and the Neurotic Triad patterns are known MMPI profiles of patients with chronic pain, and they were found for 35 of the 57 participants observed, representing over 60% of our sample. Furthermore, the High-Sensitivity group in this study included significantly more participants with the Neurotic Triad pattern than did the Others group, but it did not include any participants with the Conversion V pattern. Moreover, the scores on the Hs and Hy scales in the High-Sensitivity group fell on the border between moderate and high scores and were significantly lower than the ones in the Others group. This indicates that the participants in the High-Sensitivity group had different psychological traits from other participants with chronic pain. The lack of any significant group-to-group difference in subjective pain intensity in this study suggested that, even though no apparent differences are detected in the severity of pain reported by patients in clinical practice, their responses to QST might imply their psychological traits. Thus, assessment of pain sensitivity using the QST, which focuses on the close association between pain thresholds and psychological traits, may be particularly useful for prediction of the psychological traits of patients with chronic pain who are resistant to psychological intervention, such as psychological testing.

### Psychological traits of the high-sensitivity group

In this study, analysis of the MMPI showed that the High-Sensitivity group contained significantly more participants with the Neurotic Triad pattern, but none with the Conversion V pattern. Moreover, the scores on the Hs and Hy scales were significantly lower than those of the Others group. The Conversion V pattern relates to a characteristic profile of patients with chronic pain. It shows the tendency to replace psychological problems with physical complaints, and its socially incompatible personality is also known to cause difficulties in treatment. On the other hand, the Neurotic Triad pattern is characterized by depressive tendency and hypochondriac concerns [34]. While people with the Neurotic Triad pattern are introverted and nervous, previous studies have shown that they are less likely to engage in self-harming [29, 30] and that they respond well to multimodal treatment [57]. The Hs scale indicates a hypochondriac tendency, strong health concerns, and catastrophizing. Those who show high scores on the Hs scale tend to associate their normal physical sensations with somatic symptoms. The characteristics of the Hy scale are common to the Conversion V pattern described above. The above suggest that the High-Sensitivity group has quite different psychological aspects than the Others group.

### Limitations

In this study, data on income, social status, and working conditions of the participants were not collected, and further studies with such data will be necessary. As ethnic differences have been suggested to affect assessment of psychological traits when using the MMPI, further studies with multiple ethnic groups are needed. Since several reports have indicated that pain thresholds are affected by hormonal levels during the cycle phases [58, 59] the subjects also need to be classified by sex for analysis in future studies. Many patients with chronic pain have depression, which is known to affect pain sensation; for this reason, these patients were excluded from the study, resulting in a smaller final sample size. Furthermore, the involvement of central sensitization can be analyzed by measuring thresholds multiple times at multiple points in unaffected tissue. This study was a clinical study, thus the results might have been affected by the fact that the participants may not have been completely free from the effects of medication. However, no studies have sufficiently described the effects of drugs, such as nonsteroidal anti-inflammatory drugs and opioids, on QST [60, 61].

## Conclusions

In this study, we were able to differentiate the patients into a High-Sensitivity group by performing a cluster analysis of PTTs to two types of pain stimuli that were applied to unaffected tissue in patients with chronic pain. This High-Sensitivity group showed a significant difference in the pattern and level of neurosis scales, which is a characteristic MMPI profile of patients with chronic pain, different than the Others group. These results suggest that PTT would be a useful tool for understanding the psychological traits of patients with chronic pain.

## Abbreviations

ANOVA: Analysis of variance
MMPI: Minnesota Multiphasic Personality Inventory
PTT: Pain tolerance threshold
QST: Quantitative sensory testing
SD: Standard deviation
SF-MPQ: Short-Form McGill Pain Questionnaire
VAS: Visual analog scale
